# Women's Romantic Jealousy Predicts Risky Appearance Enhancement Effort

**DOI:** 10.1177/14747049231185782

**Published:** 2023-07-24

**Authors:** Steven Arnocky, Megan MacKinnon, Sadie Clarke, Grant McPherson, Emily Kapitanchuk

**Affiliations:** Department of Psychology, 6057 Nipissing University, North Bay, ON, Canada

**Keywords:** romantic jealousy, women's jealousy, female competition, appearance enhancement effort, attitude toward cosmetic surgery, dieting, mate retention, women's mate retention, benefit provisioning mate retention

## Abstract

Human appearance enhancement effort has recently been considered from an evolutionary perspective as an adaptive and sexually dimorphic strategy for effective female intrasexual and intersexual competition. Most writing and research on the topic to date has focused on appearance enhancement as a means of mate attraction, with relatively less research examining its role in mate retention. The present study considered whether romantic jealousy, as a negative emotion experienced in response to perceived threat to a desired relationship, predicts costly and/or risky appearance enhancement independent of the closely related emotion of envy. In a sample of 189 undergraduate women, results showed that romantic jealousy and dispositional envy were positively correlated with one another. Results further demonstrated that romantic jealousy predicted women's positive attitude toward cosmetic surgery, willingness to use a one-week free tanning membership, willingness to use a risky diet pill, and intent on spending a greater proportion of their income on appearance enhancement, but not intended use of facial cosmetics. Results held independent of participants’ dispositional envy, suggesting that romantic jealousy is a unique predictor of women's efforts at enhancing their physical appearance, which could extend into costly and physically risky mate retention efforts.

## Introduction

The progressive growth and resilience of the world's 532-billion-dollar beauty industry (Danziger, 2019) highlights a simple truth: that individuals around the world regularly attempt to enhance their physical appearance (Davis & Arnocky, 2022). Appearance enhancement effort is sexually dimorphic; compared to men, women generally engage in more costly (e.g., spending substantial time on one's appearance, using make-up, fashionable outfits, and jewelry) and potentially risky (e.g., dieting) appearance practices for the purpose of *attracting* mates (Buss, 1988a). From an adaptationist perspective, this gains some females a reproductive advantage by satisfying men's relatively greater preference for a physically attractive partner (Buss et al., 1990; Li et al., 2002). Indeed, previous research has shown that women primed with intrasexual competition for mates were more willing to use risky appearance enhancements, including a potentially dangerous diet pill and a 1-week free skin tanning membership, relative to a control group (Hill & Durante, 2011).

Further examination of individual differences among women suggests that those who are more oriented toward attracting mates (e.g., who hold a more unrestricted sociosexuality or who are higher in intrasexual competitiveness for mating opportunities) invest greater effort toward enhancing their physical appearances (Arnocky & Piché, 2014; Bleske-Rechek & Buss, 2006); this effect may be particularly enhanced among costly and risky tactics such as cosmetic surgery, rather than more benign tactics such as using facial cosmetics (Bradshaw et al., 2019). Appearance enhancement effort is also contextually driven by factors such as resource scarcity and economic uncertainty, ostensibly because these conditions increase the need to attract a male who can provide reproductively relevant resources (see Hill et al., 2012). Ultimately, both sexes rate appearance enhancement as a more effective mate attraction (Buss, 1988a) and intrasexual competition (Walters & Crawford, 1994) strategy for women.

Although considerable work has focused on the role of physical appearance enhancement in women's initial mate attraction and acquisition, much less research has considered its putative role in mate *retention*. Mate retention involves behavior aimed at preserving a valued mating relationship (Buss, 1988b; Buss et al., 2008) that is broadly motivated by romantic jealousy; the degree of emotional upset over the threatened loss of that valued relationship (Davis et al., 2018). It is presently unclear whether costly or risky appearance enhancement efforts are in fact predicted by romantic jealousy in the same manner as other mate retention tactics (see Davis et al., 2018 for review). Accordingly, the goal of this research was to determine whether romantic jealousy predicts females’ proportional spending on appearance-enhancing products and services (as an economically costly tactic), favorable attitude toward cosmetic surgery (an economically costly and physically risky tactic), and willingness to skin tan or use a potentially dangerous diet pill (physically risky tactics, Hill & Durante, 2011), relative to a more benign appearance enhancer (interest in wearing facial cosmetics) or a non-appearance-related risk variable (painting in an unventilated room).

### Appearance Enhancement as Mate Retention

Mate retention involves behavior aimed at retaining a valued mating relationship that is directed toward either intrasexual rivals (intrasexual manipulations) or one's partner (Intersexual manipulation; Buss, 1988b; Buss et al., 2008). Intrasexual manipulations aim to deter competitors from mating with one's partner and include behaviors such as public signals of possession (a way of telling potential competitors that their partner is in a relationship) and intrasexual negative inducements (direct engagement with competitors, such as threats of violence). Conversely, intersexual manipulations refer to cost inflicting or benefit provisioning behaviors aimed towards one's own partner. Whereas cost-inflicting mate retention involves behavior meant to lower a partner's self-esteem and deter rivals (e.g., becoming angry at a partner's flirting behavior), benefit-provisioning mate retention involves acts meant to increase a partner's satisfaction with the relationship (e.g., took my partner to a nice restaurant) (see Albert & Arnocky, 2016 for review). One form of benefit provisioning mate retention includes effort aimed at enhancing one's physical appearance, which can range from benign acts such as clothing choice, to more costly and risky acts such as having cosmetic surgery (Davis & Arnocky, 2022; Starratt & Shackelford, 2022).

The Mate-retention inventory (MRI; Buss, 1988a, 1988b) – a self-report measure of the diverse tactics used by men and women to retain mates, and its widely used short form (MRI-SF; Buss et al., 2008) – include a subscale of items that capture a general orientation toward enhancing one's appearance (e.g., I made myself “extra attractive” for my partner), although it does not measure specific acts of beautification (outside of two items measuring fashion choices in the long form of the measure; Buss, 1988b). Women tend to score higher on this subscale than men, suggesting that appearance enhancement is likely a female-typical mate-retention strategy that corresponds more broadly with females’ greater relative use of benefit-provisioning mate retention tactics (Buss, 1988b; Kaighobadi et al., 2010). Moreover, women in committed relationships report using appearance enhancement as a form of mate retention more than women in less committed relationships (de Miguel & Buss, 2011). However, it is presently unclear whether women rely on relatively benign acts of appearance enhancement to satisfy their mate retention goals, or whether these efforts extend to more costly and risky tactics. One study found that women's, but not men's, benefit-provisioning and cost-inflicting mate retention were associated with their interest in cosmetic surgery (Atari et al., 2017). This is not surprising given that, as described above, some items on the mate retention inventory directly assess appearance enhancement effort, potentially raising the issue of conceptual overlap between the variables that were measured. One avenue for better examining costly and risky forms of appearance enhancement as a putative form of mate retention is to determine whether romantic jealousy predicts these efforts.

### The Evolution of Jealousy and Its Role in Mate Retention

Romantic jealousy involves a suite of cognitive, emotional, and behavioral experiences surrounding the threatened loss of a valued romantic partner (e.g., White, 1981). Some researchers consider jealousy as an emotion that evolved to motivate behavior aimed at addressing specific adaptive challenges associated with infidelity and partner loss (see Arnocky et al., 2012). In this manner, jealousy fits within a broader evolutionary view of emotion as a suite of computations (based upon ecological or contextual ‘inputs’), whose ultimate function is to regulate an adaptive behavioral output (Tooby & Cosmides, 2008). Specifically, it has been argued that sexual jealousy “is engineered to prepare the body physiologically for combat, and (when the rival is weak or unwary) motivates the individual to behave violently” (Tooby & Cosmides, 2008, p. 117).

Although considerable research has focused on sex differences in its subtypes, including sexual versus emotional facets (see Buss, 2018), the overarching role of jealousy in both sexes appears to be the same: to motivate compensatory (i.e., mate retention) behavior in response to threats to a valued romantic relationship (e.g., Buss, 2000; Buss & Haselton, 2005). Much of the early research on jealousy in this domain has focused on men's sexual jealousy and partner violence (see Arnocky et al., 2012). Harris (2003) noted that research on female pathological jealousy (e.g., where suspicion of a partner's infidelity is unfounded) was lacking. Since then, research focused more specifically on women's experiences of jealousy has linked it to aggression and cost inflicting forms of mate retention. For example, Arnocky et al. (2012) found that jealousy was more prevalent among women who perceive themselves as less physically attractive than their same-sex rivals, and that jealousy mediated links to aggressive mate retention behavior. Other research has shown that women's jealousy correlated with both cost-inflicting and benefit-provisioning mate retention effort (Arnocky & Locke, 2020). Similarly, research on Iranian respondents found women's jealousy correlated with benefit-provisioning, but not cost-inflicting, mate retention (Atari et al., 2017). Davis and colleagues (2018) found that women high in reactive jealousy were more likely to engage in both cost-inflicting and benefit-provisioning mate retention, and these effects were stronger among women than men. However, it is presently unclear whether jealousy is related specifically to appearance enhancement in these studies. To date, no work has directly explored jealousy's potential role in predicting appearance enhancement effort, particularly with respect to less benign acts. Given established correlations between jealousy and benefit-provisioning mate retention more broadly, we hypothesized that jealousy would predict costly and risky forms of appearance enhancement effort. Moreover, we anticipated that this link would hold after controlling for a closely related emotion, envy, that has previously been implicated in costly and risky appearance enhancement within the context of intrasexual competition for *attracting* mates.

### Envy

Envy involves an unpleasant, negative emotional response to others who are viewed as superior in a relevant quality, or who hold a possession that is desired by the envious individual (Hill & Buss, 2006, 2008; Parrott & Smith, 1993; Smith & Kim, 2007). Envy is evoked following unfavorable social comparisons (Hill & Buss, 2008; Salovey & Rodin, 1984; Smith & Kim, 2007), and is characterized by hostility, inferiority, and resentment (Smith & Kim, 2007). Although sometimes used interchangeably, envy and jealousy are both conceptually and empirically distinct. Envy focuses on feelings of disadvantage regarding qualities or resources held by others (Smith et al., 1999), whereas jealousy focuses on threatened loss of a valued relationship (e.g., Parrott, 1991; Parrott & Smith, 1993). Research has found that individuals who were asked to recall experiences of either jealousy or envy differed in the overall quality and intensity of their affect, with jealousy appearing to be more ‘intense’ (Parrott & Smith, 1993; Salovey & Rodin, 1986). Nevertheless, there are notable similarities between these emotions. Both are rooted in interpersonal contexts and relationships, relying on social information derived from others. Envy may serve the adaptive function of alerting the individual that they are deficient, relative to intrasexual rivals, in traits or resources that are relevant to reproductive fitness (Hill & Buss, 2006, 2008; Hill et al., 2011). Because female mate value is particularly tied to their youthfulness and physical attractiveness (Buss & Barnes, 1986; Davis & Arnocky, 2022), it is not surprising that other women's attractiveness is a greater source of envy among women compared to men (DelPriore et al., 2012). For example, women reported feeling more envy when their same-sex peers became more physically attractive than them (Hill & Buss, 2006). Recently, Arnocky et al. (2016) found that envy mediated the link between upward physical appearance comparison and costly/risky appearance enhancing behavior in women, such that women higher in trait envy, and experimentally induced state envy, were more interested in spending a greater proportion of their income on appearance enhancing products and services, skin tanning, cosmetic surgery, and risky diet pill use.

### The Current Study

To date, most work on women's costly and risky appearance enhancement effort has focused on mate *attraction*, rather than retention. Conversely, work on jealousy as a predictor of mate retention has focused on broad assessments of overarching cost-inflicting or benefit-provisioning mate retention (Davis et al., 2018) or on specific cost inflicting acts, such as Intimate Partner Violence (IPV; Foran & O’Leary, 2008). Comparatively little work has considered the role that jealousy plays in predicting women's use of more specific benefit provisioning acts, including costly and risky forms of appearance enhancement effort. We hypothesized that jealousy would predict a broad spectrum of costly and/or risky appearance enhancement variables including total anticipated appearance spending on products and services, interest in using a risky diet pill known to cause health problems later in life, interest in using a one-week free tanning membership, and positive attitudes toward cosmetic surgery.

We anticipated jealousy to broadly predict a domain-general willingness to incur costs and take on risk to enhance one's appearance, and therefore we did not expect jealousy to differentially influence high cost versus high-risk variables. We also included a measure of anticipated facial cosmetics use. This variable has previously been linked to women's envy (Arnocky et al., 2016) but other research has shown facial cosmetics use to be unrelated to women's short-term mating strategies, with the authors suggesting facial cosmetics use could be a more benign act that is less situated in women's intrasexual competition (Bradshaw et al., 2019). However, other research has linked make-up usage with self-reports of intrasexual competitiveness (as well as age and mate-value), with the authors suggesting cosmetics use might serve “as a behavioral tactic of both intersexual attraction –including alteration of age perception– and intrasexual competition” (Mafra et al. 2020, p.1). From this extant literature, it is possible that if cosmetics use indicates intrasexual or intersexual mating efforts then it would likely also relate to romantic jealousy as a form of mate retention. However, it is also a more benign and less costly act that could be so normative as to not stand out as an act that is specifically tied to these motives. Therefore, we did not specify a hypothesis for this variable in relation to jealousy but rather considered its inclusion as exploratory. Further, we hypothesized that these links would remain independent of the effects of envy on these variables. Jealousy and envy have a long history of being conflated in everyday use, and in both clinical and academic practice (see Spielman, 1971), prompting studies aimed at distinguishing between the two (Parrott & Smith, 1993). Accordingly, a secondary goal of this research was to examine the extent to which romantic jealousy and dispositional envy were positively correlated with one another.

## Method

### Participants and Procedure

In the fall of 2019 as part of a larger study on health and mating behavior, undergraduate women were recruited using the institutional research system at Nipissing University located in North Bay, Ontario, Canada. Participants were 189 mostly Caucasian (91%) females aged 17–37 (*M_age_* = 20, *SD* = 2.64). Approximately half (55%; *n* = 104) were in a committed romantic relationship at the time of data collection, with an average length of one year. Participants received partial credit.

### Procedure and Measures

Participants provided written informed consent and were led to a private room where they completed the following self-report measures:

#### Jealousy

The Multidimensional Jealousy Scale (MJS) was used to assess cognitive, emotional, and behavioral jealousy (Pfeiffer & Wong, 1989). Participants were told to think of their current or most recent serious romantic relationship and respond to each statement on a seven-point Likert-type scale of 1 = *Never/Very Pleasant* to 7 = *All of the time/Very upset* (Pfeiffer & Wong, 1989). Example items include: “How often do you have the following thoughts about X?: I suspect that X is secretly seeing someone of the opposite sex” (Cognitive), “How would you emotionally react to the following situations?: X is flirting with someone of the opposite sex” (Emotional), and “How often do you engage in the following behaviors?: I question X about previous or present romantic relationships” (Behavioral). Because we did not have specific predictions about sub-facets of jealousy and given that each component contributes to the overall experienced emotion of jealousy, following previous research (e.g., Arnocky & Locke, 2020; Lantagne & Furman, 2017; Maner et al., 2007) we created a mean score by averaging responses across all items. The measure showed good internal consistency, (α = .88).

#### Dispositional Envy

The Dispositional Envy Scale (DES) measures individual differences in envy (Smith et al., 1999). The DES assesses individuals’ experience of perceived inferiority and experienced frustration in response to someone else's advantage, and subjective feelings of injustice. The DES includes 8 items ranked on a 7-point Likert-type scale ranging between 1 = *strongly disagree* and 7 = *strongly agree*. An example item is: “Feelings of envy constantly torment me”. The items were averaged to create a dispositional envy score, which demonstrated good internal consistency, (α = .87).

#### Cosmetic Surgery

Attitude toward cosmetic surgery was measured using the 15-item Acceptance of Cosmetic Surgery Scale (ACSS; Henderson-King & Henderson-King, 2005). Participants indicated the extent to which they agree or disagree with statements reflecting acceptance of cosmetic surgery on a 7-point Likert-type scale, ranging from 1 = *Strong Disagreement*, 7 = *Strong Agreement* (Henderson-King & Henderson-King, 2005). The measure contains three components; an intrapersonal factor (self-oriented beliefs surrounding cosmetic surgery), a social factor (social motivators to have cosmetic surgery) and a consideration factor (whether they would have the surgery or not) (Henderson-King & Henderson-King, 2005). Example items include “It makes sense to have minor cosmetic surgery rather than spending years feeling bad about the way you look” (Intrapersonal), “If a simple cosmetic procedure would make me more attractive to others than I would think about trying it” (Social), and “If I could have a surgical procedure done for free, I would consider trying cosmetic surgery” (Consideration; Henderson-King & Henderson-King, 2005). Because we did not have a-priori predictions about specific subscales, we followed previous research (Arnocky & Piché, 2014; Arnocky et al., 2016) considering overall mean scores across the full measure as a favorable attitude toward cosmetic surgery. The measure showed good internal consistency, (α = .90).

#### Appearance Enhancement Products

The proportion of income participants spent on consumer goods and services aimed at enhancing their physical appearance was measured using an 11-point scale from 0% to 100% (with 10% intervals). A list of example products was provided: clothing, make-up, jewelry, hair styling, haircuts, cologne/perfume, and skin treatments/products (Arnocky et al., 2016). Participants reported spending an average of approximately 25% of their income on such products/services.

#### Tanning and Diet Pill Use

Participants endorsed their degree of interest in using a free tanning membership, taking a diet pill known to cause heart problems later in life, and a control risk-taking variable of willingness to paint in a room that is not properly ventilated to avoid outside noise and inclement weather (Hill & Durante, 2011). The control variable served to determine if a participant's risk-taking behaviors were specific to appearance enhancement. Each item was rated on a 7-point Likert-type scale, ranging from 1 = *not at all interested* or *never* to 7 = *very interested* or *very frequently* (Hill & Durante, 2011).

*Cosmetics use.* The Situational Cosmetics Use Inventory (Cash & Walker-Cash, 1982) examined intended use of facial cosmetics in everyday situations. Using a 5-point Likert-type scale ranging from 0 = *no cosmetics use* to 4 = *use as much as ever used in any situation*, participants rated their intended cosmetics use in eight social situations ranging from spending the day indoors reading or doing things along to attending a formal wedding reception. The measure showed good internal consistency, (α = .89).

### Data Analysis Plan

An observed variable path model examined links between relationship status, romantic jealousy, and the risky appearance enhancement variables, as well as the risk control item of painting in an unventilated room. We anticipated that current relationship status may correlate more strongly with jealousy, and thus would serve as a control variable in the model. We further anticipated that jealousy and envy would correlate with one another, and would independently predict each costly or risky appearance variable, but not the control risk variable of painting in an unventilated room. We also included exploratory links to facial cosmetics attitude, with the prediction that envy (but perhaps not jealousy) would predict intended use. We expected some of the appearance-enhancement outcomes to correlate with one-another, so we used the bivariate correlation table to eliminate paths between uncorrelated outcomes or control variables (i.e., relationship status) prior to running the model. Path analysis is more suitable than ordinary least-squares regression for such analyses because as it can be used to test jealousy's predictive relation to observed appearance enhancement variables simultaneously, while controlling for their intercorrelation with one another. Missing data were minimal (< 5%) and a regression imputation (AMOS 28) was used following examination of the descriptives and bivariate correlations (Table 1). Good model fit was indicated by a non-significant model *χ^2^*, the comparative fit index (CFI; > .90), the normed fit index (NFI; > .90), and the root mean square error of approximation (RMSEA; < .80) (Kline, 2005).

## Results

Descriptive statistics and bivariate correlations among study variables are presented in Table 1. Currently being in a heterosexual romantic relationship did not correlate with any variables in the model and was thus dropped from the subsequent path model.^1^

**Table 1.** Descriptive Statistics and Correlations Between Study Variables Using Raw Data Prior to Missing Values Regression Imputation for Path Analysis.

	M	SD	Min.	Max.	1	2	3	4	5	6	7	8
1. Relat. Status.	—	—	—	—	—							
2. Jealousy	2.05	1.29	1.00	7.00	.10	—						
3. Envy	3.35	1.17	1.00	7.00	.13	.29***	—					
4. Appear. Spend.	24.71	17.54	0.00	80.00	.05	.21**	.09	—				
5. Skin Tan	2.90	2.07	1.00	7.00	−.11	.24***	.14	.19**	—			
6. Diet Pill	1.66	1.29	1.00	7.00	.05	.33***	.29***	.02	.26***	—		
7. Risk Control	3.12	2.11	1.00	7.00	−.02	.07	.14	.13	−.03	.01	—	
8. Cosmetic Surg.	2.94	1.13	1.00	5.93	−.06	.26***	.23**	.21**	.26***	.20**	.13	—
9. Cosmetics use	1.60	0.70	0.00	3.75	−.12	.10	.12	.10	.11	−.01	.10	.32***

Relationship status: 1 = ‘No’, 2 = ‘Yes’. *p* < .05. ** = *p* < .01. *** = *p* < .001.

**Figure 1.**
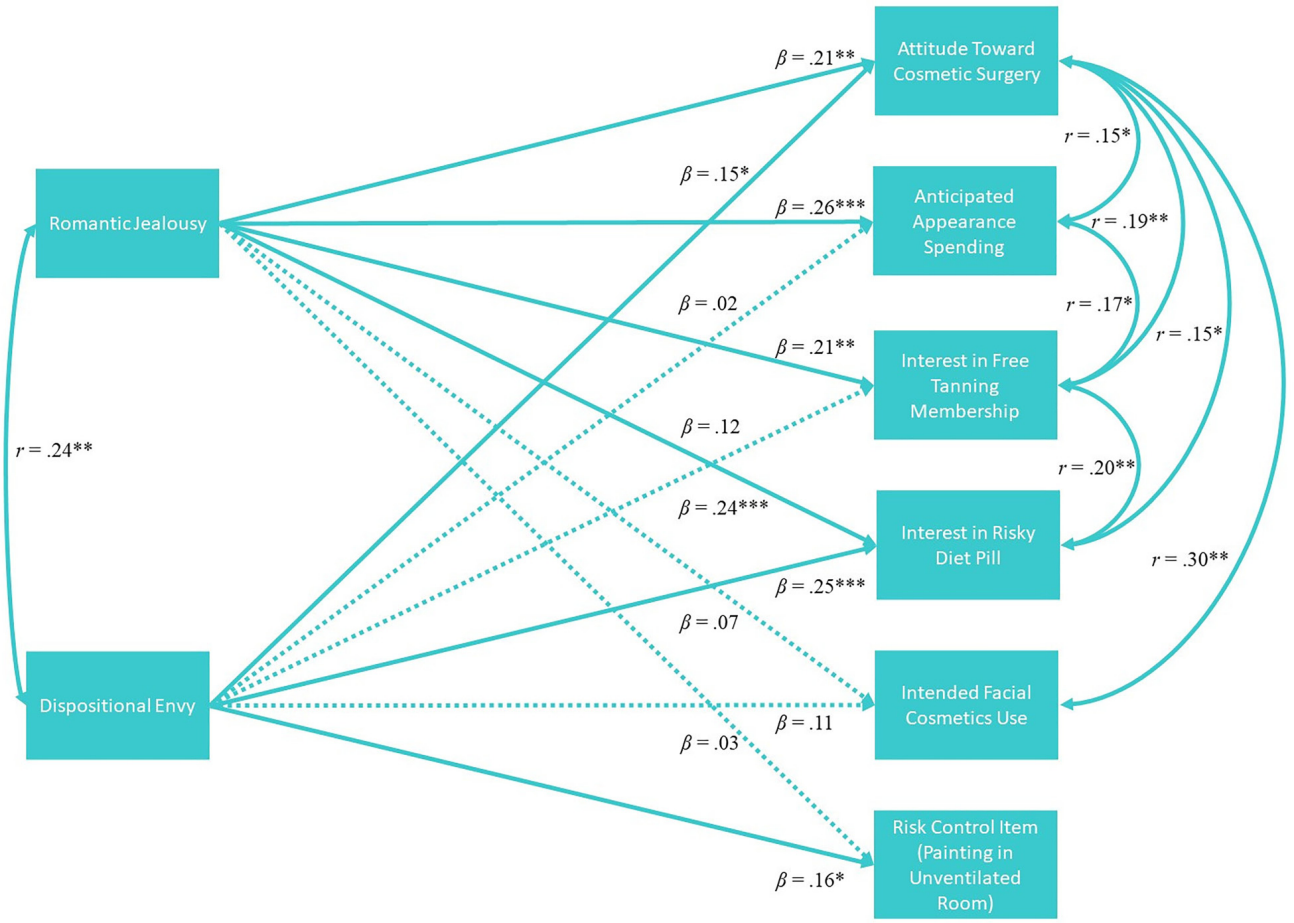
Results of observed variable path analysis demonstrating links between jealousy and physical appearance enhancement attitudes.

Next, we tested a path model that examined whether jealousy predicted each measure of risky appearance enhancement effort, controlling for the effects of envy, which is closely related to jealousy and has been shown to predict many of the same appearance enhancement variables (Arnocky et al., 2016). We used the bivariate correlation matrix to trim initial null covariances between the five risky appearance enhancement dependent variables, as well as the non-significant relationship between envy and intended appearance spending (Figure 1). Results showed that envy and jealousy were positively correlated (*r* = .24, *p* = .001). Similar to previous research (Arnocky et al., 2016), envy predicted most appearance enhancement variables: Specifically, envy predicted attitude toward cosmetic surgery (*b* = 0.02, SE = .01, *β* = .15, *p* = .04), willingness to take a risky diet pill (*b* = 0.05, SE = .01, *β* = .25, *p* < .001), and modestly predicted intended tanning membership use (*b* = 0.03, SE = 02, *β* = .12, *p* = .097), but not appearance spending (*b* = 0.01, SE = 02, *β* = .02, *p* = .82) or facial cosmetics use (*b* = 0.07, SE = 04, *β* = .11, *p* = .13). Jealousy predicted all of the appearance enhancement outcome variables: anticipated appearance spending (*b* = 0.81, SE = .23, *β* = .26, *p* < .001), positive attitude toward cosmetic surgery (*b* = 0.31, SE = .11, *β* = .21, *p* = .004), willingness to take a risky diet pill (*b* = 0.49, SE = .14, *β* = .24, *p* < .001), and willingness to use a free tanning membership (*b* = 0.56, SE = 20, *β* = .21, *p* = .004). Jealousy did not predict facial cosmetics use (*b* = 0.44, SE = 10, *β* = .07, *p* = .33), or the control risk variable of painting in an unventilated room, suggesting that jealousy-linked risk-taking was specific to enhancing appearance (*b* = 0.08, SE = .20, *β* = .03, *p* = .70). The model fit the data well, *χ*^2^ = 5.13 (*df* = 8, *p* = .74), RMSEA = 0.00 (95% CI = 0.000–0.060), CFI = 1.00, NFI = .96, AIC = 77.13.^2^^,^^3^

## Discussion

Men prefer youthful and physically attractive partners (Buss, 1989; Buss & Barnes, 1986; Buss et al., 1990); a preference that appears to be more strongly held by men who view themselves as high in mate value (Arnocky, 2018) or who experience cues to high resource holding potential (such as holding large sums of money, Yong & Li, 2012). Appearance enhancement behavior has therefore been considered from an evolutionary perspective as a means of outcompeting intrasexual rivals for attracting desirable mates (see Davis & Arnocky, 2022 for review). Beyond mate attraction, some work has also considered appearance enhancement broadly as one form of benefit provisioning mate retention – a means by which individuals can entice their partners to remain in the relationship (Buss et al., 2008). The brief items on measures of mate retention dealing with appearance enhancement are typically endorsed more by women than by men (Buss et al., 2008). However, it is presently unclear whether jealousy, as an emotion known to underlie both cost-inflicting and benefit provisioning mate retention, indeed predicts risky and costly appearance enhancement acts independent of the closely related emotion of envy, which itself has been shown to motivate women's risky and costly appearance enhancement within the context of outcompeting same-sex rivals.

Results of the present study largely replicated previous research showing that dispositional envy predicted (or trended toward) most forms of appearance enhancement effort, except for anticipated appearance product and service spending over the next month. Results also showed that jealousy predicted all measures of appearance enhancement effort: positive attitude toward cosmetic surgery, anticipated appearance enhancing product and service spending, willingness to use a risky diet pill, willingness to use a skin tanning membership, but not intended facial cosmetics use. The finding that appearance spending was tied to jealousy but not envy suggests that it may be more relevant for intersexual competition (specifically mate retention) versus pure intrasexual competition. Some research has shown that as women age (up to their 40's) they increase their spending on clothing relative to early adulthood spending (see Lutz, 2013 for review), even though their status has increased, and many have presumably secured a mate. This could indicate a drive for workplace competitive success (looking appropriate at work) or, perhaps, as a means of retaining one's partner. Future research should explore whether jealousy maps on to individual differences in the trajectory of women's appearance spending as they age and develop long-term mating relations.

Moreover, jealousy did not predict the control risk taking variable of painting in an unventilated room, suggesting that jealous women are not generally prone to risk taking, but rather that jealousy-related risk taking was specific to appearance enhancement in this study, supporting our hypothesis. Another interesting finding was that jealousy did not predict intended facial cosmetics use. Other recent researchers (Bradshaw et al., 2019) have similarly observed that women high in short-term mating effort were more likely to endorse risky appearance procedures such as cosmetic surgery, but not in their use of facial cosmetics, which they argued to be a relatively low-cost appearance enhancement technique. Based on these findings, it is possible that a competitive mating orientation in women may specifically predict more costly and risky appearance enhancement efforts, as opposed to lower risk, benign or normative acts.

Results also demonstrated that dispositional envy and jealousy were moderately (*r* = .29) correlated with one another in the path model. To our knowledge, this is the first study to demonstrate a statistical relationship between these constructs. Some researchers have considered envy as a specific sub-case of jealousy (e.g., Salovey & Rodin, 1984; 1986), whereas others have highlighted important conceptual distinctions between them (Parrott & Smith, 1993). Our findings suggest that jealousy and envy are related both to one another, and to some common forms of fitness enhancing effort (e.g., appearance enhancement effort), yet are simultaneously distinct constructs with potentially divergent evolutionary roots. Perhaps envy is more relevant to motivation within the realm of intrasexual selection, whereas jealousy is an emotion that is more relevant to intersexual selection. The close relationship between these emotions appears to mirror the fact that intrasexual and intersexual competition are intertwined, such that effort aimed at attracting and retaining a desirable mate also removes that mate as a prospective partner for same sex rivals. The findings that jealousy was uniquely related to appearance enhancement when controlling for envy highlights the utility of exploring these emotions as distinct empirical constructs, given that they may relate differentially to some fitness-enhancing behaviors within the realm of sexual competition. For example, if envy is more closely linked to intrasexual competition and jealousy to intersexual competition, we might see meaningful differences in the targets of interpersonal aggression. Perhaps envy better predicts aggression toward members of the same sex, whereas romantic jealousy may better predict aggression toward one's romantic partner.

These findings have implications for the study of appearance enhancement from an evolutionary perspective as a broad strategy for improving reproductive fitness by enhancing one's ability to attract *and* retain desirable mates. Moreover, these findings provide further empirical support for the consideration of jealousy as an adaptive emotion that evolved to motivate compensatory behavior in response to threats to a valued relationship. Most previous work on jealousy has focused on its links to negative, cost-inflicting acts of mate-retention, such as intimate partner violence (Buss, 2000). This research highlights that jealousy also plays a role in predicting benefit provisioning acts, such as appearance enhancement, aimed at enticing one's partner to remain in the dyad, even when those acts are costly or risky to the actor.

### Limitations and Future Directions

There were several limitations to this research which would benefit from future work examining the link between jealousy and appearance enhancement. First, our sample was relatively homogenous in both age and ethnicity. Because we were interested in assessing jealousy and appearance effort in a sample where both attraction effort and mating competition are high, we limited our sample to young reproductive-aged women. We relied on a WEIRD (Western Educated Industrialized Rich Democratic) sample, which could also influence the perpetration of cost inflicting versus benefit provisioning mate retention. Previous work has linked lower socioeconomic status to some indices of cost inflicting mate retention, such as domestic violence perpetration by men and women (Bhona et al., 2019; Wilt & Olson, 1996). Similarly, economic recession and uncertainty has been linked to women's appearance enhancement effort (e.g., Hill et al., 2012). Thus, it is unclear as to whether the results would replicate or perhaps strengthen in a more diverse sample, such as with broader socioeconomic statuses among participants. It would be interesting to examine whether these results hold in non-heterosexual samples. VanderLaan and Vasey (2007) found that homosexual women's mate retention appeared sex atypical. Perhaps, then, researchers might expect the links observed here between jealousy and appearance enhancement to be weaker or non-existent among a sample of homosexual females. Similarly, research would benefit from considering potential links between jealousy, envy, and *men's* appearance enhancement. Although women engage on more appearance enhancement for the purposes of mate attraction and retention (Buss, 1988b), men also engage in appearance enhancement for mate attraction and retention purposes (see Davis & Arnocky, 2022). It is also possible that jealousy might predict more male-typical tactics, such as pursuit of status and resources within the context of mate retention (Buss, 1988b).

It is possible that because the MJS incorporates a behavioral subscale within the measure, that subscale could be artificially inflating the link between the MJS and other jealousy-related behavior. To address this, we re-ran our final model with the behavioral subscale removed from the composite MJS measure (α = .87). Results did not meaningfully change from those reported herein, suggesting that appearance enhancement effort was predicted independently by a composite measure of cognitive (i.e., perceiving a threat to the relationship) and emotional jealousy. The utility of the MJS for participants who are presently single should also be considered. Because singly participants completed the measure in reference to their most recent relationship, it is difficult to know how this might affect their mate retention motivations. Rather, it is possible that single participants’ mate attraction motivations were primed by reflecting on a relationship they are no longer in, which could result in both single and partnered women reporting interest in appearance enhancement, resulting in a null correlation between relationship status and the appearance enhancement variables. However, it should be noted that controlling for relationship status did not meaningfully alter our model, and post-hoc analyses restricting the model to only women currently in a romantic relationship revealed jealousy as a predictor of all appearance enhancement variables except for diet pill use (which was the only variable that envy predicted). This suggests that jealousy was a robust predictor of appearance effort, supporting its putative role in mate retention.

It is possible that the nature of the MJS measure could inadvertently prime participants to experience an increase in state jealousy, simply by having completed the measure. Accordingly, it is unclear whether the jealousy captured in this study was indicative of trait or state levels of jealousy, or perhaps some combination of each. Regardless, the model tested in this study involving jealousy as a predictor of appearance related effort should apply to both trait and state experiences. Future research would benefit from distinguishing between these factors by focusing more experimentally on the state-based experience of jealousy and subsequent shifts in appearance effort (e.g., attitudes, intentions, or in-vivo behavior).

This research was correlational in nature, and therefore, the causal role of jealousy in appearance enhancement cannot be ascertained. Previous work on envy has used experimental priming tasks to induce the emotion and measure subsequent attitudes and intentions toward risky and costly appearance enhancement practices (Arnocky et al., 2016). Previous research has used jealousy induction techniques whereby participants imagine a partner committing an infidelity. It is possible that the directional interpretation of jealousy as an emotion that motivates behavior in response to a reproductive threat is incorrect. For instance, jealousy and commonly associated behavior could be simultaneously activated in response to adaptive challenges as related features of a single information-processing and behavior-regulating system, or as two separate systems that become concurrently activated because they happen to have a common ‘input’ variable, such as suspected infidelity or threatened loss of partner. Future research could use this same priming task to induce jealousy (versus a control group) and subsequently examine appearance enhancement attitudes and intentions. This would better examine the directional role of jealousy in motivating compensatory behavior (in this case, appearance enhancement) in response to an adaptive challenge (imagined infidelity) in line the with evolutionary perspective on emotion. It would also be interesting to go beyond self-reports of appearance enhancement attitudes and intent to examine behavioral efforts in this domain. For instance, researchers could offer as remuneration for study participation gift cards that are focused on appearance enhancement (skin tanning, cosmetics store, aesthetics salon) versus non-appearance gift cards (groceries, entertainment). This would eliminate any potential social desirability effects associated with appearance enhancement measures.

Future research should also consider relationship satisfaction in relation with these variables. First, it would be interesting to determine whether appearance enhancement effort is an effective form of mate retention. Previous work has generally supported the notion that such efforts (e.g., cosmetic surgery) can improve others’ rating of one's physical attractiveness, but whether this has meaningful longitudinal implications for effective mate retention remains to be seen. Second, it is possible that state relationship satisfaction might moderate the links observed in this study, such that jealous individuals who are simultaneously satisfied with their partner might be more motivated to engage in appearance enhancement effort than jealous women who are less satisfied in their relationship.

## Conclusions

Recently, researchers have considered appearance enhancement efforts from an evolutionary perspective as a means of enhancing one's reproductive fitness. Previous research on women has highlighted the role of appearance enhancement effort in intrasexual competition and mate attraction, but relatively less work had considered its role in mate retention. The present study found that romantic jealousy predicted a constellation of costly and risky appearance enhancement attitudes and intentions. These findings suggest that women are more likely to exert appearance enhancement effort when they perceive a threat to a valued relationship, and that the emotion of jealousy may motivate costly and risky appearance enhancement behaviors.

## Notes

1. Including relationship status as a predictor of each variable in the model did not meaningfully change any of the results reported herein.

2. Previous research has identified that appearance enhancement may be more frequently used as a mate retention tactic by heterosexual versus homosexual women. Eliminating non-heterosexual participants from the sample (*n *= 22) did not meaningfully alter any reported results.

3. As part of the larger survey, we had included a brief mate value (MV) in the survey (the Mate Value Scale, Edlund & Sagarin, 2014). Including self-reported MV as a covariate into the model did not meaningfully alter the findings or model fit reported herein. MV negatively correlated with envy but was unrelated to jealousy. MV correlated positively with skin tanning intent, but negatively with willingness to use the diet pill.
